# Supplementary material for: Phenotypic and Molecular Characterization of Multidrug-Resistant Clinical Isolates of the Candidozyma haemuli Species Complex (Formerly Candida haemulonii Species Complex) from the Brazilian Amazon Reveals the First Case of Candidozyma pseudohaemuli in Brazil
Source: J Fungi (Basel). 2025 May 20;11(5):394. doi: 10.3390/jof11050394 (PMC12113443; doi:10.3390/jof11050394)
Supplement: Supplementary file 1 [file jof-11-00394-s001.zip › jof-3607071-supplementary.pdf]

Supplementary Table S1: Biofilm Biomass and Metabolic Activity in *Candidozyma haemuli* species complex and reference strains.*

Isolate	Species	Biofilm mass					Metabolic activity				
		Tripicate					Tripicate				
		1°	2°	3°	Mean	Standart deviation	1°	2°	3°	Mean	Standart deviation
IEC-CAND1	*C. duobushaemuli*	0,392	0,193	0,327	0,30	0,10	0,374	0,301	0,622	0,43	0,17
IEC-CAND9	*C. duobushaemuli*	0,618	0,466	0,388	0,49	0,12	0,513	0,355	0,497	0,46	0,09
IEC-CAND29	*C. duobushaemuli*	0,200	0,116	0,265	0,19	0,07	0,193	0,181	0,069	0,15	0,07
IEC-CAND30	*C. duobushaemuli*	0,193	0,158	0,138	0,16	0,03	0,124	0,313	0,083	0,17	0,12
IEC-CAND35	*C. duobushaemuli*	0,166	0,121	0,093	0,13	0,04	0,123	0,083	0,068	0,09	0,03
IEC-CAND97	*C. duobushaemuli*	0,326	0,296	0,215	0,28	0,06	0,247	0,102	0,277	0,21	0,09
IEC-CAND108	*C. duobushaemuli*	0,192	0,232	0,317	0,25	0,06	0,158	0,143	0,177	0,16	0,02
IEC-CAND248	*C. duobushaemuli*	0,192	0,247		0,22	0,03	0,123	0,175	0,183	0,16	0,03
IEC-CAND270	*C. duobushaemuli*	0,246	0,115	0,222	0,19	0,07	0,182	0,306	0,252	0,25	0,06
IEC-CAND305	*C. duobushaemuli*	0,261	0,355	0,266	0,29	0,05	0,207	0,207	0,246	0,22	0,02
IEC-CAND312	*C. duobushaemuli*	0,436	0,365	0,386	0,40	0,04	0,398	0,256	0,414	0,36	0,09
IEC-CAND6	*C. haemuli*	0,494	0,342	0,362	0,40	0,08	0,481	0,411	0,333	0,41	0,07
IEC-CAND8	*C. haemuli*	0,327	0,455	0,422	0,40	0,07	0,324	0,31	0,47	0,37	0,09
IEC-CAND10	*C. haemuli*	0,301	0,212	0,205	0,24	0,05	0,316	0,268	0,255	0,28	0,03
IEC-CAND17	*C. haemuli*	0,625	0,361	0,468	0,48	0,13	0,361	0,305	0,335	0,33	0,03
IEC-CAND119	*C. haemuli*	0,372	0,315	0,266	0,32	0,05	0,373	0,246	0,383	0,33	0,08
IEC-CAND199	*C. haemuli*	0,320	0,335	0,313	0,32	0,01	0,309	0,491	0,411	0,40	0,09
IEC-CAND254	*C. haemuli*	0,255	0,314	0,354	0,31	0,05	0,325	0,396	0,446	0,39	0,06
IEC-CAND286	*C. haemuli*	0,213	0,210	0,211	0,21	0,00	0,309	0,467	0,334	0,37	0,08
IEC-CAND306	*C. haemuli*	0,068	0,080	0,061	0,07	0,01	0,312	0,525	0,48	0,44	0,11
IEC-CAND314	*C. haemuli*	0,170	0,264	0,170	0,20	0,05	0,134	0,244	0,169	0,18	0,06
IEC-CAND301	*C. haemuli var. vulneris*	0,267	0,419	0,348	0,34	0,08	0,143	0,29	0,24	0,22	0,07
IEC-CAND307	*C. haemuli var. vulneris*	0,169	0,186	0,199	0,18	0,02	0,139	0,012	0,069	0,07	0,06
IEC-CAND49	*C. pseudohaemuli*	0,162	0,245	0,309	0,24	0,07	0,328	0,209	0,234	0,26	0,06
IEC-CAND290	*C. albicans*	0,148	0,152	0,201	0,17	0,03	0,093	0,107	0,039	0,08	0,04
ATCC_22019	*C. parapsilosis*	0,144	0,233	0,204	0,19	0,05	0,053	0,121	0,038	0,07	0,04
ATCC_C.auris	*C. auris*	0,240	0,160	0,177	0,19	0,04	0,593	0,32	0,294	0,40	0,17

* Each isolate was tested in triplicate for biofilm formation and metabolic activity. The table shows individual replicate values, means, and standard deviations for both biofilm mass (measured by crystal violet assay) and metabolic activity (measured by XTT reduction assay).
